# Boosting Organic Solar Cell Performance via Light-Assisted Crystallization of P3HT:PCBM Blend

**DOI:** 10.1021/acsomega.5c03436

**Published:** 2025-07-23

**Authors:** Duygu Akın Kara, Sevdiye Basak Turgut, Burak Gultekin

**Affiliations:** Solar Energy Institute, 37509 Ege University, 35000 Izmir, Turkey

## Abstract

In this study, we explore the effect of halogen-light-assisted (LA) annealing on the crystallization of poly­(3-hexylthiophene) (P3HT) films and the morphology of P3HT:[6,6]-phenyl-C61-butyric acid methyl ester (PCBM) blends, along with their impact on organic solar cell performance. Samples were annealed at various temperatures (100–120 °C) and durations (5–15 min) using both conventional hot plate (HP) and LA methods. Structural analysis showed that LA annealing significantly enhanced the crystallinity of P3HT films through improved in-plane molecular stacking. For the P3HT:PCBM blend layers, the best film quality and device performance were achieved with LA annealing at 100 °C for 15 min, resulting in a power conversion efficiency (PCE) of 3.10% compared to 2.22% for the HP-annealed counterpart. We further evaluated blends with different P3HT:PCBM ratios (1:0.6, 1:1, and 1:1.4) and found that P3HT-rich compositions, especially 1:0.6, benefited most from LA annealing, achieving better film morphology and higher efficiency. Charge transport and recombination behavior were studied through time-resolved photoluminescence, light-intensity-dependent *J*–*V*, and space charge limited current measurements. These analyses confirmed that LA annealing improves charge carrier mobility, reduces trap densities, and minimizes nonradiative recombination. Overall, our results demonstrate that LA annealing is an effective and scalable method to enhance both the material quality and photovoltaic performance in P3HT:PCBM-based solar cells.

## Graphical abstract

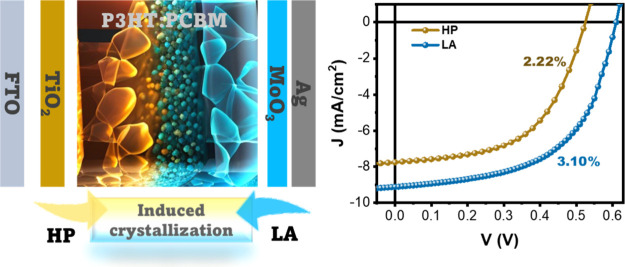

## Introduction

Organic solar cells have received remarkable attention as a cost-effective and flexible alternative to traditional silicon-based solar cells.^1–3^ Among various designs, the bulk heterojunction (BHJ) architecture, which consists of two or more materials with complementary electron affinities such as the conjugated polymer poly­(3-hexylthiophene) (P3HT) and the fullerene derivative phenyl-C61-butyric acid methyl ester (PCBM), is widely researched and considered promising.^4,5^ The morphology of the active layer, consisting of donor and acceptor components, is a key factor in improving the efficiency of solar cells.^6^ P3HT efficiently absorbs visible light and transports holes, while PCBM is an efficient electron acceptor. P3HT:PCBM blends formed interpenetrating nanoscale domains, facilitating efficient charge separation and transport.^7^ The effectiveness of the BHJ structure, called the active layer, depends on the morphology and material properties. Thermal annealing of P3HT can cause crystallization, which can significantly impact the morphology of the active layer in P3HT:PCBM BHJ solar cells.^8^ P3HT crystallization can increase its ordering and charge-transport capabilities, but excessive crystallization can cause aggregation and creation of massive crystalline domains, which can impede charge transport and decrease device efficiency.^9^ Additionally, the thermal annealing process can result in phase separation between P3HT and PCBM, resulting in a suboptimal morphology and a negative impact on device performance.^10,11^ Besides, thermal annealing of P3HT in BHJ solar cells is a critical process that can significantly impact the stability and reproducibility of devices.^12^ Hence, careful optimization of annealing conditions is necessary to achieve optimal morphology of the active layer, improved interfaces, and enhanced performance.^13^ Also, the other important point which affects the kinetics of P3HT crystallization and PCBM aggregation with their correlations is the ratio of P3HT and PCBM components in the blend (P3HT-rich, PCBM-rich, and equimolar) introducing morphological variation of P3HT/PCBM composite films.^14,15^ During the photovoltaic process, the molecular structure of the donor or acceptor phases directly impacts the light absorption and carrier mobility. The magnitude of phase separation between the donor and acceptor phases regulates the surface area of the donor–acceptor interface at which excitons dissociate, and the distance excitons need to spread to reach an acceptor.^16^ Therefore, the active layer shape significantly influences the photovoltaic device performance.^17^ The formation of crystals in the P3HT phase suggests better charge separation at the interfaces with PCBM. On the other hand, a method named photoannealing has been conducted with the presence of a halogen lamp, benefiting from exciting trap carriers by photons with high energy and releasing the space charge carriers. Therefore, the activation energy of the active layer crystallization is formed by these high-energy photons. In addition, light-assisted (LA) annealing enhances the molecular ordering of P3HT chains due to the synergistic effects of radiative heating and photon-induced excitation. The interaction of high-energy photons (in the visible and near-infrared spectrum) with the polymer matrix not only raises the local temperature but also provides additional energy to overcome kinetic barriers for π–π stacking, thereby promoting a better alignment of P3HT backbones. This ordering is supported by the enhanced vibronic shoulders in the UV–vis absorption spectra and the more intense (100) diffraction peaks observed in the X-ray diffraction (XRD) analysis.^18^ Inversely, infrared radiation with low energy photons provides crystallization in the thermal (conventional) annealing procedure.^19^ During thermal annealing, further crystallization is prevented by Coulomb repulsion in the space charge region, which is formalized from trap states. LA or the other name of photoannealing procedure led to improved crystallinity, reduced trap density, and enhanced performance.^20^ Moreover, the Scherrer analysis confirmed that LA-annealed films possess crystallite domains larger than those of their hot plate (HP)-annealed counterparts. Unlike slow bulk heating in HP, LA provides more localized and controlled heating from the film surface inward, enabling rapid reorganization of polymer chains while avoiding overgrowth or thermal degradation.^21^

Herein, two different annealing methods, thermal annealing via HP and LA annealing via halogen lamp, have been carried out to explore the crystallinity of P3HT and blend film morphology to increase the charge carrier mobility and power conversion efficiency (PCE). We have utilized a different annealing process consisting of varied annealing temperatures and durations via HP and LA procedures to obtain the optimum annealing way for high-quality P3HT:PCBM film morphology. Moreover, using an optimal annealing temperature and time, we focused on the P3HT:PCBM ratios in the blend to understand the LA-annealing procedure on PCBM and P3HT domains separately. For this purpose, P3HT:PCBM in different compositions were placed on top of FTO/TiO_2_ layers in organic BHJ solar cells. This study aims to explore the annealing techniques used in P3HT:PCBM-based solar cells, investigate how light affects the active layer, and understand how light affects the device performance. To evaluate the effect of the LA process on the structure and morphology of P3HT and P3HT:PCBM blend layers, XRD, atomic force microscopy (AFM), photoluminescence (PL) spectroscopy, and UV–vis absorption spectroscopy techniques were carried out. The changes caused by thermal annealing under various conditions and the effect of the annealing process on the solar cell devices were evaluated by using a range of optoelectrical characterization methods. Specifically, a study has reported a maximum PCE of 4.17% using high-molecular-weight P3HT without the need for postprocessing treatments such as thermal annealing or solvent additives.^22^ Similarly, in another work by Wang et al., P3HT:PCBM devices processed with different blend ratios achieved PCEs of 2.8 (1:2) and 3.5% (1:3) depending on the composition.^4^ Considering these reports, our PCE value of 3.10% obtained via a simple LA method, without using additives or complex interfacial engineering, falls well within the competitive range and demonstrates the effectiveness and practicality of our approach.

## Results and Discussion

### Thin Film Characterizations

We investigated the different annealing temperature and duration effects upon P3HT film crystallization under HP and LA-annealing procedures. According to XRD results, P3HT-coated films exhibited one dominant peak at 5.20° associated with the (100) plane representing thiophane rings of P3HT resulting in first-order reflection also corresponding to well-organized and oriented structures. Increased annealing temperature (from 100 to 120 °C) under conventional annealing has improved the intensity of these peaks indicating better crystallinity of P3HT, whereas there is no significant effect on peak intensity in terms of rising temperature in LA-annealed films.^23,24^ Regarding the same annealing temperature (100 °C) for different annealing times (5, 10, and 15 min) for both annealing procedures, the crystallographic planes of P3HT films have been investigated. Figure S1a illustrates that LA-annealed films exhibited much greater peak intensity explained by in-plane stacking of P3HT chains than the conventional annealed counterparts at the same annealing duration.^25^ Also, one can notice that, in the HP annealing procedure, the annealing time affected the P3HT crystallization directly, and the best film crystallization was achieved at 100 °C for 15 min. On the other hand, LA-annealed films exhibited almost the same peak intensity, and thus a short annealing duration (5 min) is sufficient to get good crystallization under this type of annealing procedure. In this respect, optical absorption and PL spectra were investigated (Figure S1b,c). The presence of the shoulder peak around 600 nm in the absorption spectrum indicates the ordered and aligned P3HT molecules. Increased temperature and time lead to improved absorbance intensity which is an indicator of the P3HT chain ordering for both annealing procedures.^26^ On the other hand, all LA-annealed films exhibited enhanced absorption intensity and broadening which is helpful for light harvesting, giving rise to improved photovoltaic efficiency.^27^ According to the PL response of P3HT films, the P3HT PL spectrum typically exhibits two peaks at room temperature, which are usually assigned to the (0–0) and (0–1) transitions between vibrational energy levels in the literature.^28^ In there, the (0–0) peak at 664 nm (1.86 eV) is less intense than the (0–1) peak centered at 730 nm (1.70 eV) in the PL data spectrum. As shown in Figure S1c, with increasing annealing time in films annealed with HP, a decrease in the PL intensity can be interpreted, leading to a reduction in P3HT clustering. Although a similar behavior is observed on surfaces annealed with LA, a conflict of systematic shifting between the film annealed at 100 °C for 10 min and 120 °C for 5 min is attributed to the annealing method, specifically the slower annealing of surfaces, resulting in fewer trap states and balanced reduction in recombination due to reduced trap density.^29^ However, it is not sufficient to interpret the crystal structure of P3HT solely on the basis of PL. High emission intensities were observed in the thin films of P3HT annealed at 100 °C HP and LA for 5 min, and the emission line widths are broader compared to the films annealed at HP and LA 100 °C for 15 min. The film annealed via LA for 15 min has lower trap states compared to the film annealed in HP for the same duration.^30^ Therefore, the emission results agree with the XRD data. To understand the effect of different annealing times and temperatures by using different annealing procedures on the P3HT surface morphology, we have used the AFM technique. In Figure S2, higher annealing temperature and longer time increased the crystallinity of P3HT domains employing both HP and LA-annealing procedures. In addition, the XRD results of P3HT confirm that the crystal peak of the LA film is more distinct and has a higher intensity; also, the AFM images support that the LA film has better crystallinity. In light of these findings, 100 °C for 15 min was chosen as the optimum annealing temperature and duration of P3HT for both HP and LA.

### P3HT:PCBM Film

Similar to P3HT film characterizations, including XRD, AFM, and optical measurements, the effect of annealing procedures on P3HT:PCBM (1:1) BHJ films has been investigated. As shown in Figure S3, in the XRD spectra of BHJ films, the same trend was observed with P3HT film crystallinity due to annealing time, temperature, and thermal procedure. LA-annealed surfaces exhibited a higher intensity of peak at 5.20° corresponding to P3HT crystal domains indicating enhanced formation and organization of P3HT chains in comparison with conventional thermally annealed layers under the same temperature and duration.^31^ In the blend film, after thermal annealing, PCBM molecules diffuse into the P3HT matrix, enabling the latter to undergo reorganization into stacks and long chains due to the expansion of mean free volume in the presence of LA procedure.^32^ Improved organization and crystallization of the films enable them to have higher mobility and enhanced device performance.^33^ According to calculated crystallite sizes by Scherrer equation, LA-annealed films showed higher crystallinity values of 10.87 nm than HP-processed ones of value 7.93 nm for 100 °C, 15 min.^34^ Table S1 summarizes the effects of various annealing methods and conditions on the grain size. Notably, LA at 100 °C for 15 min resulted in the largest crystallites, suggesting enhanced molecular ordering compared to HP-treated counterparts under similar or shorter durations.^35,36^

During morphological investigations, we observed that AFM images of the P3HT:PCBM films showed that the annealing time increases phase separation and aggregation (Figure S4). Preferred phase distribution such as the enrichment of donors at the anode and acceptors at the cathode represents an optimal morphology for lower charge recombination and improvement in charge collection efficiency.^37^ LA-annealed films exhibited more crystalline and homogeneously dispersed nanostructure of P3HT and PCBM forms at each temperature and time.^38^

Figure 1a illustrates the absorption spectrum of P3HT:PCBM (1:1) BHJ films annealed with various annealing procedures. One main and two shoulder absorption peaks were observed at around 520, 560, and 620 nm, respectively. The first peak is associated with P3HT main chains, and the other vibronic peaks are related to the extension of the P3HT conjugation length (560 nm) and the transition between P3HT chains (620 nm).^39^ P3HT:PCBM films which are LA-annealed at 100 °C for 15 min exhibited the highest peak intensity and broader wavelength leading to good arrangement in the P3HT conjugated chains which is associated with enhanced charge-transport properties and exciton dissociation of BHJ solar cells.^40^

**Figure 1:**
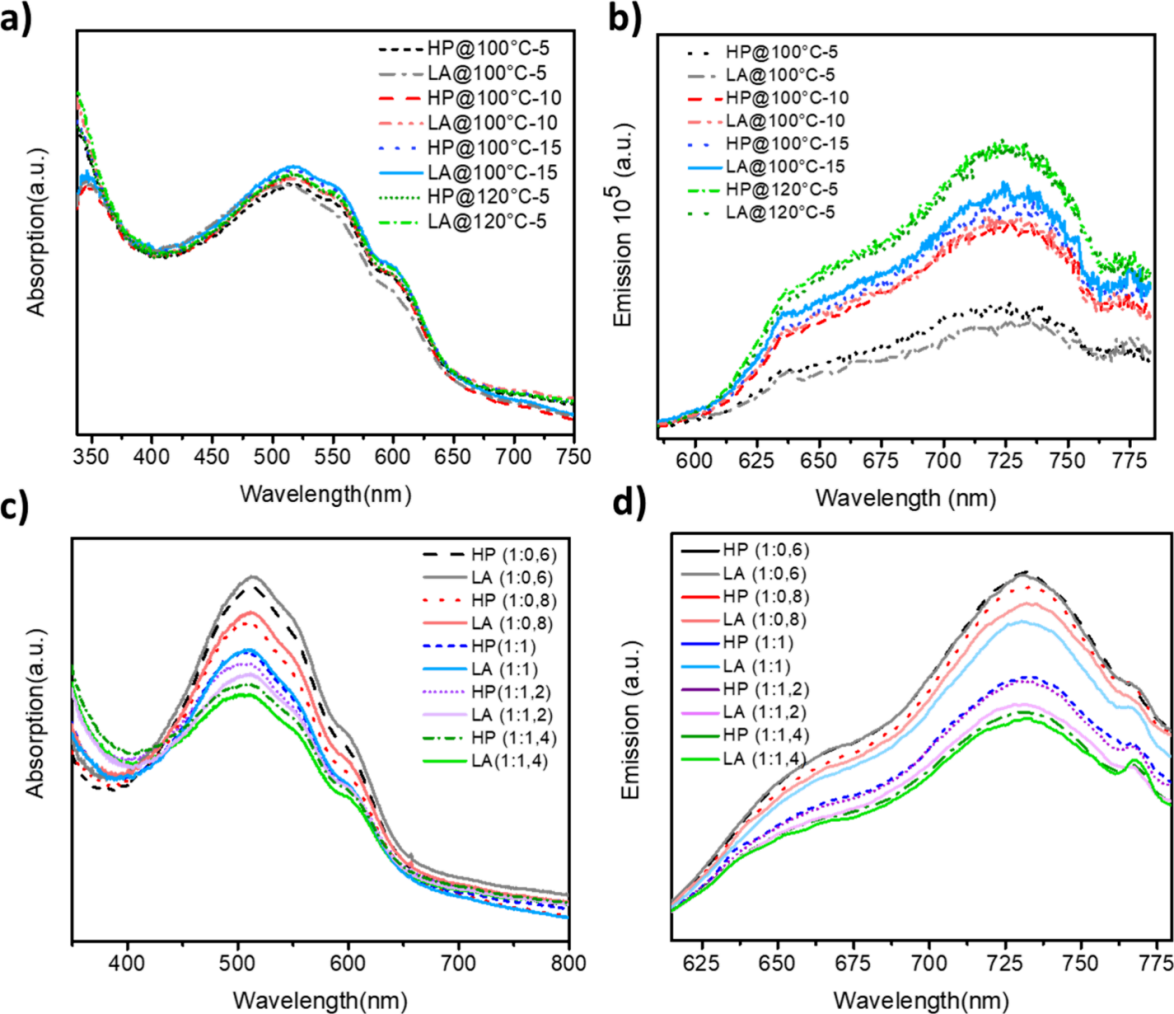
(a, b) Absorbance and emission spectra of P3HT:PCBM (1:1) BHJ film annealed at various temperatures and times with different annealing procedures and (c, d) absorbance and emission spectra of P3HT:PCBM BHJ film with various ratios of PCBM which is annealed at 100 °C for 15 min with different annealing types.

The films annealed at 100 °C for 5 min presented a lower absorption peak intensity by using both annealing procedures, causing less ordering in the thin film. In connection with the result, LA- and HP-annealed films have the same peak intensity representing main P3HT crystallinity, but LA-annealed ones showed lower peak intensity between 550 and 650 nm resulting in a lower degree of conjugation and interchain transition.^22^ Enhanced morphology can be attributed to the reorganization of the polymer molecules and nanoparticles. Through annealing, the structural integrity of the P3HT polymer chains, previously compromised by the presence of PCBM domains, is restored by separating PCBM molecules from the polymer matrix. This annealing-induced movement within both P3HT and PCBM entities prompts their reconfiguration, resulting in the formation of a phase-separated three-dimensional architecture comprising donor and acceptor molecules.^41^ Figure 1b shows the PL spectra of the P3HT:PCBM (1:1) BHJ films. Here, the blend film with LA-annealed at 100 °C for 15 min illustrated narrower spectra introducing better crystallinity and formation of the blend film, whereas 120 °C for 5 min exhibited broader and higher PL peaks. P3HT:PCBM films annealed under both procedures of 100 °C for 10 min and 100 °C for 5 min via HP and LA showed lower intensity and broader PL spectra while representing pure molecular reorganization and phase separation resulting in worse morphology.^42^ According to PL results, LA-annealed films with a process of 100 °C for 15 min showed higher PL intensity and narrower spectra in comparison with HP-annealed ones.

### Different P3HT:PCBM Blend Ratios

For the next step, we discussed the different annealing procedure effects on the BHJ film with different PCBM ratios. Initially, we fixed the annealing temperature and time and investigated LA annealing in the formation on the film regarding P3HT and PCBM domains separately. For this purpose, we have chosen the annealing procedure at 100 °C for 15 min since the maximum absorbance has been achieved with this type of procedure for both HP- and LA-annealed equimolar (1:1) BHJ films. In Figure 1c, it can be noticed that the increasing P3HT rate gives rise to a higher absorption peak and shifts to wider wavelengths. LA-annealed P3HT-rich (1:0.6 and 1:0.8) films exhibited stronger absorption spectra than the films with conventional annealing. Increased characteristic peak of the π–π * transition of the P3HT backbone due to the enhanced π–π stacking of the P3HT molecule resulted in higher chain ordering. Otherwise, PCBM-rich (1:1.2 and 1:1.4) films presented weaker absorption spectra in comparison to thermal annealing. HP-annealed films exhibited higher peak intensity in both P3HT and PCBM parts since the LA annealing has no positive effect on PCBM-rich films, as much as P3HT-rich films can result from degradation of PCBM in the presence of the LA procedure.^43^ For equimolar films (1:1), there is no big difference in the absorption spectrum like PCBM or P3HT-rich films, but still, LA-annealed counterparts showed higher absorption peak intensity, confirming the results seen in Figure 1a. Thus, we can say that the LA-annealing process amends the P3HT formation, especially the degree of chain crystallinity and conjugation.^44^ Figure 1d illustrates a gradual decrease in the PL emission intensity with increasing PCBM concentration in the blend film, representing improved efficiency of charge transfer. A clear trend of decreasing PL intensity is observed with increasing PCBM content, which indicates more effective exciton dissociation and charge transfer between donor and acceptor phases. This PL quenching behavior is particularly noticeable in P3HT-rich blends (e.g., 1:0.6), where the partial suppression of emission suggests an optimized donor–acceptor interface that facilitates efficient hole transfer from PCBM to the P3HT phase. Reduced PL intensity correlates with improved exciton separation and lower radiative recombination, confirming enhanced charge-transfer efficiency at the molecular level. This also aligns with the observed increase in hole mobility and reduced trap density in the same composition, indicating that LA-annealed P3HT-rich blends not only support better morphology but also more effective electronic coupling between donor and acceptor domains. Additionally, it refines the surface morphology and interconnects the polymer chains, thus augmenting hole mobility within the polymer network.^45^ AFM images of P3HT:PCBM films annealed via the LA and HP procedure of 100 °C for 15 min can be seen in Figure S5. P3HT-rich and equimolar blend films show better homogeneous surfaces and lower RMS than PCBM-rich films. In addition, LA annealing appears to regulate the phase separation between P3HT and PCBM more favorably. The AFM images reveal that LA-treated blend films exhibit finer and more uniform domains, which can be attributed to the controlled diffusion of PCBM and reorganization of the polymer network. This controlled phase morphology helps maximize the donor–acceptor interfacial area while preventing the formation of charge-blocking PCBM-rich aggregates. Consequently, LA annealing significantly reduces the trap density in both P3HT and P3HT:PCBM films. This is evidenced by longer carrier lifetimes in time-resolved photoluminescence (TRPL) measurements and lower trap-filled limit voltages derived from space charge limited current (SCLC) analysis. The decrease in nonradiative recombination and the enhanced carrier mobility suggest that the LA process effectively passivates trap states and creates a cleaner, more ordered electronic environment.^21,41^

Together, these findings support the conclusion that LA annealing is not merely a thermal process but a combined radiative and photonic treatment that synergistically modifies the molecular structure, electronic landscape, and phase morphology, thereby leading to enhanced solar cell performance.

### Current Voltage Characterizations

The effect of the annealing procedure on the performance of BHJ organic solar cells with the FTO/TiO_2_/ P3HT:PCBM/MoO_3_/Ag structure has been investigated. The current density–voltage (*J*–*V*) characteristics of devices have been realized under an AM1.5 solar simulator source of 100 mW/cm^2^. Figure 2a shows the *J*–*V* characteristics of P3HT:PCBM (1:1) devices with different annealing procedures with various annealing temperatures and durations. The best performance has been observed by the P3HT:PCBM device by LA annealing at 100 °C for 15 min with a PCE of 3.10%, *J*_SC_ of 9.11 mA/cm^2^, *V*_OC_ of 610 mV, and FF of 0.55, whereas the same device annealed with conventional thermal annealing exhibited lower performance with a PCE of 2.22%, *J*_SC_ of 7.75 mA/cm^2^, *V*_OC_ of 520 mV, and FF of 0.56. Photovoltaic parameters of the devices are summarized in Table 1. According to the solar cell parameters, LA-annealed devices exhibited higher *V*_OC_ and *J*_SC_ than the HP ones resulting in decreased nonradiative recombination and improved charge carrier transport.^46^ Moreover, there exists a significant relationship between *V*_OC_ and charge separation. The impact of charge delocalization on charge separation is closely linked to the annealing procedure. Lower charge separation and reduced electron–hole separation distances in charge-transfer states decrease charge separation, resulting in lower *V*_OC_. This phenomenon occurs particularly when annealing conditions are insufficient, leading to diminished *V*_OC_ values.^47^ Figure 2b shows the statistical graph of the P3HT:PCBM (1:1) BHJ cell performance under different temperatures for both annealing procedures. In there, HP-annealed devices showed more reproducible results, but the efficiencies are not as high as with LA-processed samples. This could be a result of bulk film discrepancy in the case of formation of crystallization.^48^ Also, for each temperature, a statistical chart graph depicting the *V*_OC_ change in both directions with 10 devices is provided in Figure S6. Due to the chart graphs of reproducibility, similar behavior with efficiency has been observed. Figure 2c,d illustrates the hysteresis behavior of HP and LA inducted devices, respectively, with typical current density–voltage (*J*–*V*) under forward (from −0.1 to 1 V) and reverse (from 1 to −0.1 V) scan characteristics of the optimized devices. Hysteresis behavior has been investigated for the annealing procedure on charge accumulation during the solar cell operation. In two types of devices, inverted hysteresis has been conducted, meaning that reverse scan results show less *J*_SC_ and *V*_OC_ which occurred due to charge accumulation at the interfaces and surface recombination of the active layer.^49^ The most noticeable decrease in the *V*_OC_ values are approximately 70 and 20 V for HP- and LA-processed devices, respectively. The lower decrease of *V*_OC_ in LA-annealed ones is explained by the decrease in surface recombination and lowered traps. In addition, reduced hysteresis has been obtained for LA-annealed cells associated with improved charge collection as well as decreased charge accumulation and defect cites.^50^

**Figure 2:**
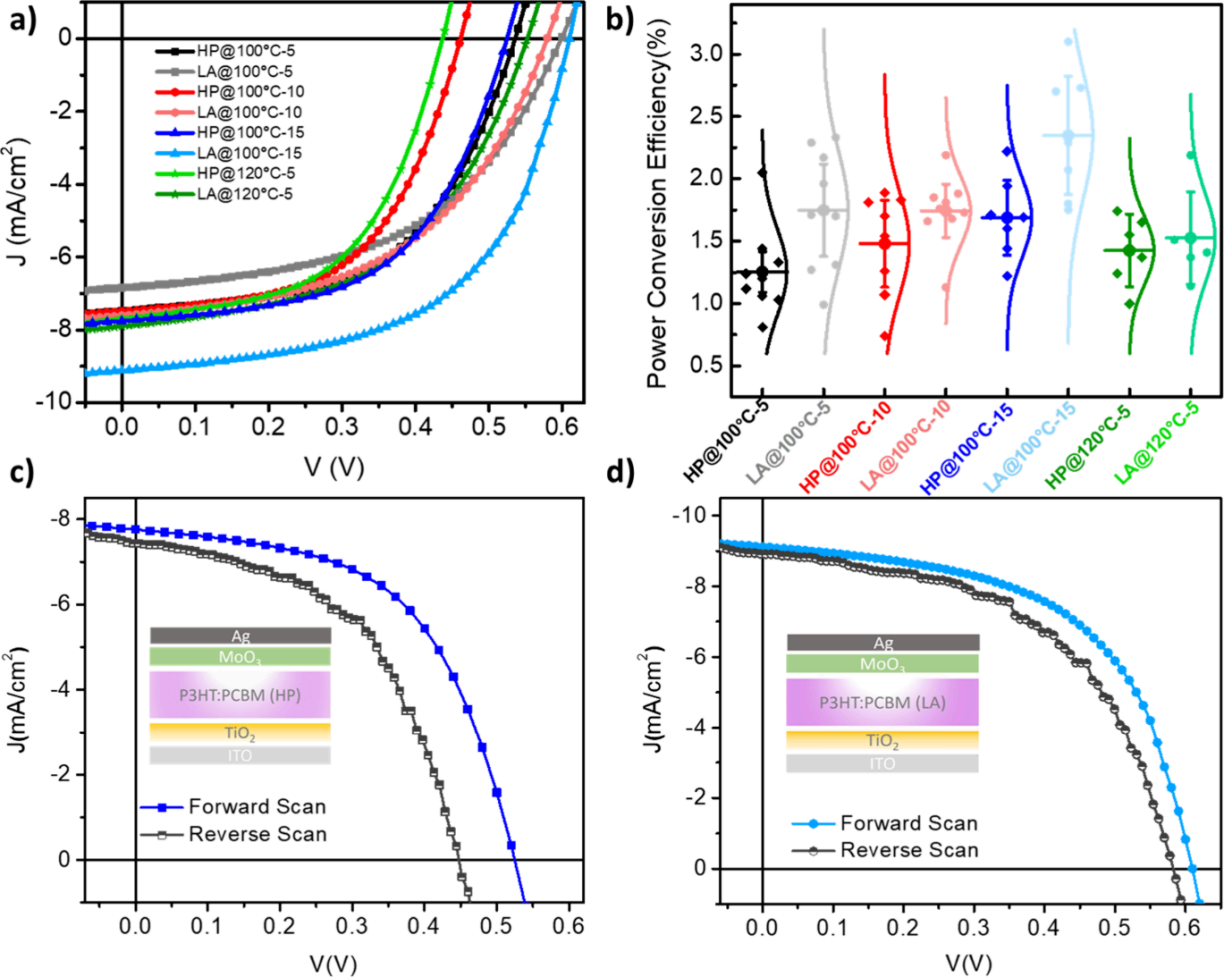
(a) *J*–*V* characteristics of devices with LA and HP annealing under different temperatures and durations. (b) PCE variation for different annealing procedures. (c and d) Forward (0–1.1 V) and reverse (1.1–0 V) scan directions under illumination at 300 K of devices with HP and LA annealing under 100 °C for 15 min, respectively.

**Table 1:** Photovoltaic Parameters of Devices with LA and HP Annealing at Different Temperatures and Durations

sample	*J*_SC_ (mA/cm^2^)	*V*_OC_ (mV)	FF	PCE (%)
HP@100 °C-5	7.56	470	0.48	1.70
LA @100 °C-5	6.84	600	0.50	2.05
HP@100 °C-10	7.48	460	0.54	1.89
LA @100 °C-10	7.60	580	0.49	2.19
HP@100 °C-15	7.75	520	0.55	2.22
LA@100 °C-15	9.11	610	0.55	3.10
HP@120 °C-5	7.68	440	0.53	1.80
LA @120 °C-5	7.89	550	0.50	2.19

So far in this work, the goal of finding the most effective annealing procedure has been achieved, with 100 °C for 15 min, yielding the best results for both HP and LA methods. In the subsequent section, annealing temperature and duration were kept constant, and the ratio of PCBM in the P3HT:PCBM mixture has been varied, investigating the light-induced effect on PCBM-rich or P3HT-rich mixture. The main objective was to comprehend the impact of different PCBM ratios on the LA procedure, elucidating the positive or negative changes on P3HT or PCBM films and corroborating the information obtained in the previous section.^51^ To achieve this purpose, the 1:1 ratio was initially considered as the reference point for our deeper fundamental investigations. Then, BHJ mixtures enriched with P3HT and PCBM at ratios of 1:0.6 and 1:1.4 were employed in BHJ solar cells.

In Figure 3a, the current density–voltage (*J*–*V*) characteristics of different types of devices fabricated via LA or HP procedure have been investigated under an AM 1.5 solar simulator source of 100 mW/cm^2^. According to these results, in BHJ solar cells with an excess of P3HT, it is evident that there is a significant disparity between LA and HP procedures, with devices produced through the LA procedure exhibiting higher device parameters and better PCE in comparison with those of HP-processed ones. The other point is that P3HT-domain-rich devices exhibited the highest current density and saturation, showcasing superior performance when compared to other ratios (Table 2) which is also a confirmation of absorption and emission spectra in terms of various PCBM concentrations. Figure 3b illustrates the incident photon- to-current conversion efficiency (IPCE) spectra which represents the ability of the cell to convert light to electric current of the fabricated P3HT:PCBM solar cells with different PCBM ratios under LA and HP procedures.^34^ As seen, devices with a P3HT-rich blend show dramatic enhancement under both annealing procedures in comparison to equimolar and PCBM-rich mixtures. On the other hand, P3HT-rich devices fabricated under the LA procedure exhibited a stronger IPCE peak at the wavelength of 550 nm than the same BHJ devices under the HP procedure, resulting from recombination reduction.^35^ To verify the positive effect of the LA procedure on the reproducibility of device performance, we have analyzed the P3HT-rich BHJ device data from 10 optimized solar cells.

**Figure 3:**
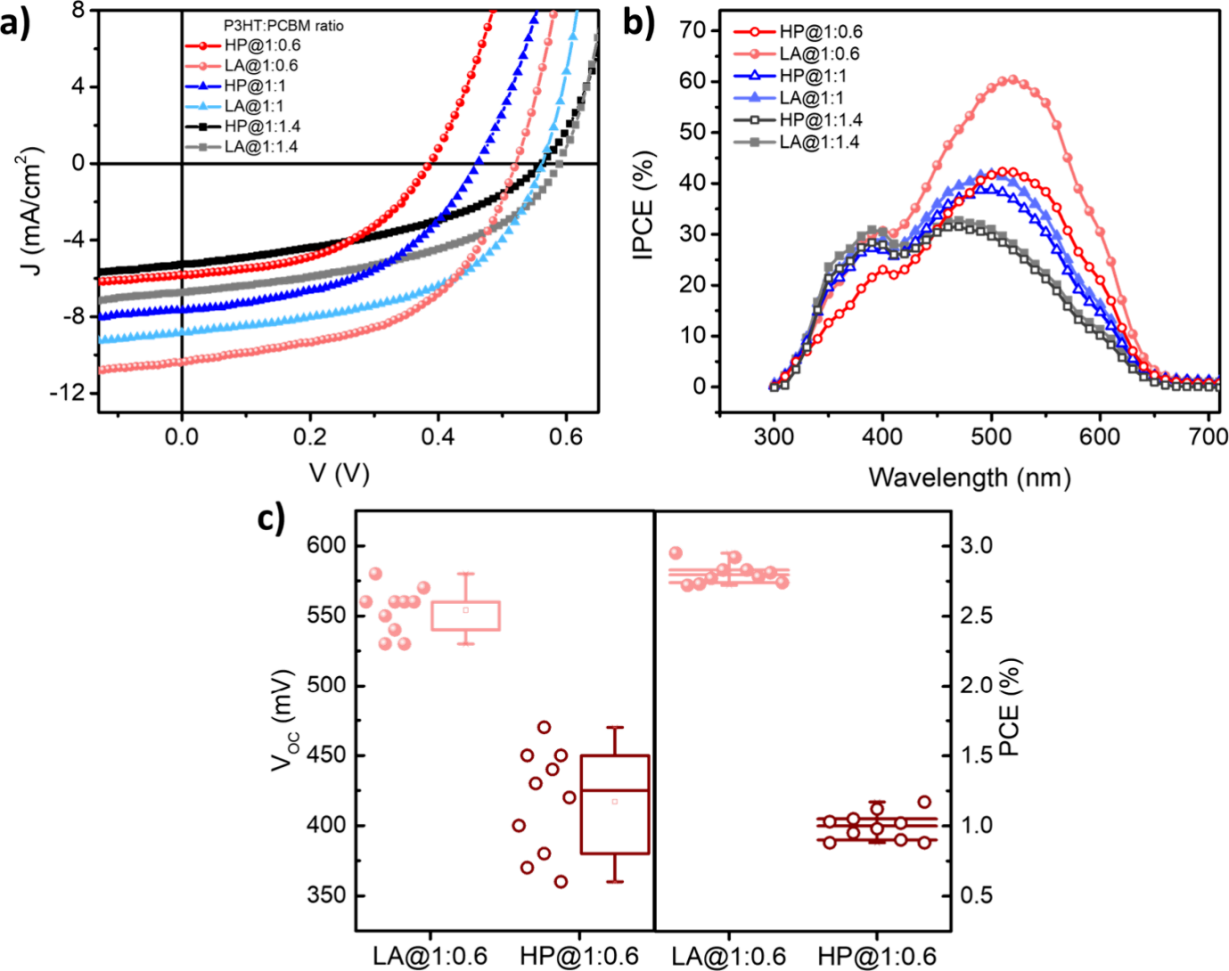
(a) *J*–*V* characteristics, (b) IPCE spectra, and (c) performance statistics including distribution of *V*_OC_ and PCE (%) of devices with different PCBM ratios under LA and HP annealing procedures (100 °C for 15 min).

**Table 2:** Photovoltaic Parameters of Devices with P3HT:PCBM Blend in Different Compositions Annealed via HP and LA Procedures (100 °C for 15 min)

sample	*J*_SC_ (mA/cm^2^)	*V*_OC_ (mV)	FF	PCE (%)
HP@1:0.6	–5.93	400	0.48	1.12
LA@1:0.6	–9.98	540	0.54	2.92
HP@1:1	–7.71	460	0.47	1.64
LA@1:1	–8.82	580	0.53	2.67
HP@1:1.4	–5.4	570	0.41	1.24
LA@1:1.4	–7.0	600	0.44	1.87

Figure 3c represents the statistical results including the distribution of *V*_OC_ and PCE data for the cells fabricated with HP and LA procedures on box chart graphics. *V*_OC_ data distribution of devices fabricated with the LA procedure was quite narrower than that of the fabricated devices with the HP procedure. Besides, PCE data distributions of cells are almost similar; however, still the cells fabricated with the LA procedure show closer values representing that higher reproducibility has been achieved.

### Charge-Transport Properties

To understand the carrier recombination mechanism, TRPL is one of the widely used methods.^52^ In Figure 4, TRPL curves are displayed by a biexponential fitting which consists of a fast component (τ_1_) and a slowest component (τ_2_). τ_1_ is the shortest lifetime attributed to nonradiative recombination proceeding from defect or trap states, and τ_2_ corresponds to intrinsic radiative recombination.^53^ Meanwhile, Figure 4 shows the TRPL decay and fitting of glass/P3HT:PCBM indicating the photoexcited carrier lifetimes (τ_1_ and τ_2_) of P3HT:PCBM films fabricated in different compositions (1:0.6, 1:1, and 1:1.4) via HP or LA procedure. Figure 4a illustrates the different annealing processes’ effect upon the TRPL result of P3HT-rich blend films. P3HT:PCBM films fabricated with the HP procedure exhibit a carrier lifetime τ_1_ = 0.24 ns and τ_2_ = 2.58 ns (*X* ^2^ = 1.08 which is the fitting parameter) shorter than the lifetime of LA-processed films of τ_1_ = 0.28 and τ_2_ = 2.73 ns (*X* ^2^ = 1.26). Extended PL lifetime represents the reduced existing traps in the bulk film and surface states at the crystal boundaries caused by disordered polymer molecular chains.^54,55^ A similar phenomenon has also been observed in other P3HT:PCBM films with different PCBM ratios led by two annealing procedures. In Figure 4b, the TRPL response of the P3HT:PCBM blend with a 1:1 mixture can be seen. According to this result, photoexcited charge lifetimes increased the presence of LA procedure to τ_1_ = 0.27 ns and τ_2_ = 2.54 ns (*X* ^2^ = 1.08) from the lifetime of τ_1_ = 0.25 and τ_2_ = 2.50 ns (*X* ^2^ = 1.09) films with the HP procedure.^56^ Figure 4c shows the PL results of PCBM-rich P3HT:PCBM (1:1.4) blend. Both LA and HP annealing procedures have almost the same shortest lifetime τ_1_ = 0.30 ns and quite close slowest components 2.35 (*X* ^2^ = 1.12) and 2.41 ns (*X* ^2^ = 1.35), respectively. These results show that charge carrier recombination was suppressed in LA-processed P3HT:PCBM films with all blends, leading to improved device performance.^57^ A more pronounced difference in the carrier lifetime between LA- and HP-treated devices has been observed in the P3HT-rich compositions. This observation is further corroborated by the current–voltage characteristics, indicating a consistent correlation between the photophysical behavior and the overall device performance. To further investigate the charge carrier extraction and recombination mechanisms affecting the device performance with the presence of the LA-type annealing procedure and varied blend compositions, we have studied the light intensity-dependent *J*–*V* characterizations. In this part of the work, *J*_SC_ and *V*_OC_ values were extracted from the current density–voltage characteristics of devices under various light intensities which are changed from 1 to 100 mW/cm^2^. During this investigation, we used two basic methods such as *V*_OC_-log light intensity and log *J*_SC_-log light intensity giving insights about detailed recombination types.^58^ Figure 5a– c shows the slope of the semilogarithmic plot of *V*_OC_ versus light intensity graph introducing the charge recombination mechanism and can be calculated by the following eq 1.^59^

$$\frac{\partial V_{\mathrm{OC}}}{\partial \ln I}=n\frac{k_{B}T}{q}$$ 1

where *q* is the electron charge, *k*_B_ is the Boltzmann constant, *T* is the temperature in Kelvin, *I* is the intensity of incident light, and *n* is the ideality factor. In this sense, *n* = 1 represents bimolecular recombination which is the dominant mechanism in operating solar cells, whereas a larger value of *n* (*n* > 1) indicates the presence of monomolecular recombination in addition to bimolecular recombination.^60^ In Figure 5a, *n* values were found to be 1.44 and 1.60 *k*_B_ *T*/*q* for LA- and HP-processed P3HT-rich P3HT:PCBM (1:0.6) solar cells, respectively. According to this result, it can be said that charge recombination realized in devices fabricated via the LA procedure was radiative recombination on a large scale instead of trap-assisted (monomolecular) recombination in comparison with devices fabricated via the HP procedure.

**Figure 4:**
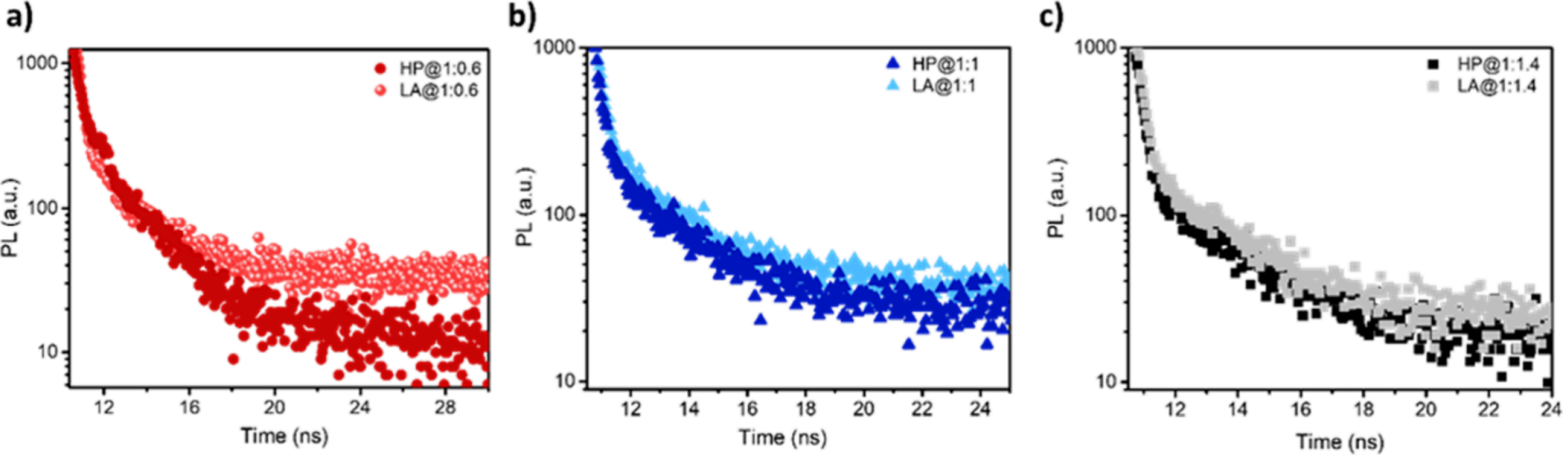
TRPL decays and fitting data of glass/P3HT:PCBM films fabricated in different compositions of (a) 1:0.6, (b) 1:1, (c) 1:1.4 in different annealing procedures HP and LA.

**Figure 5:**
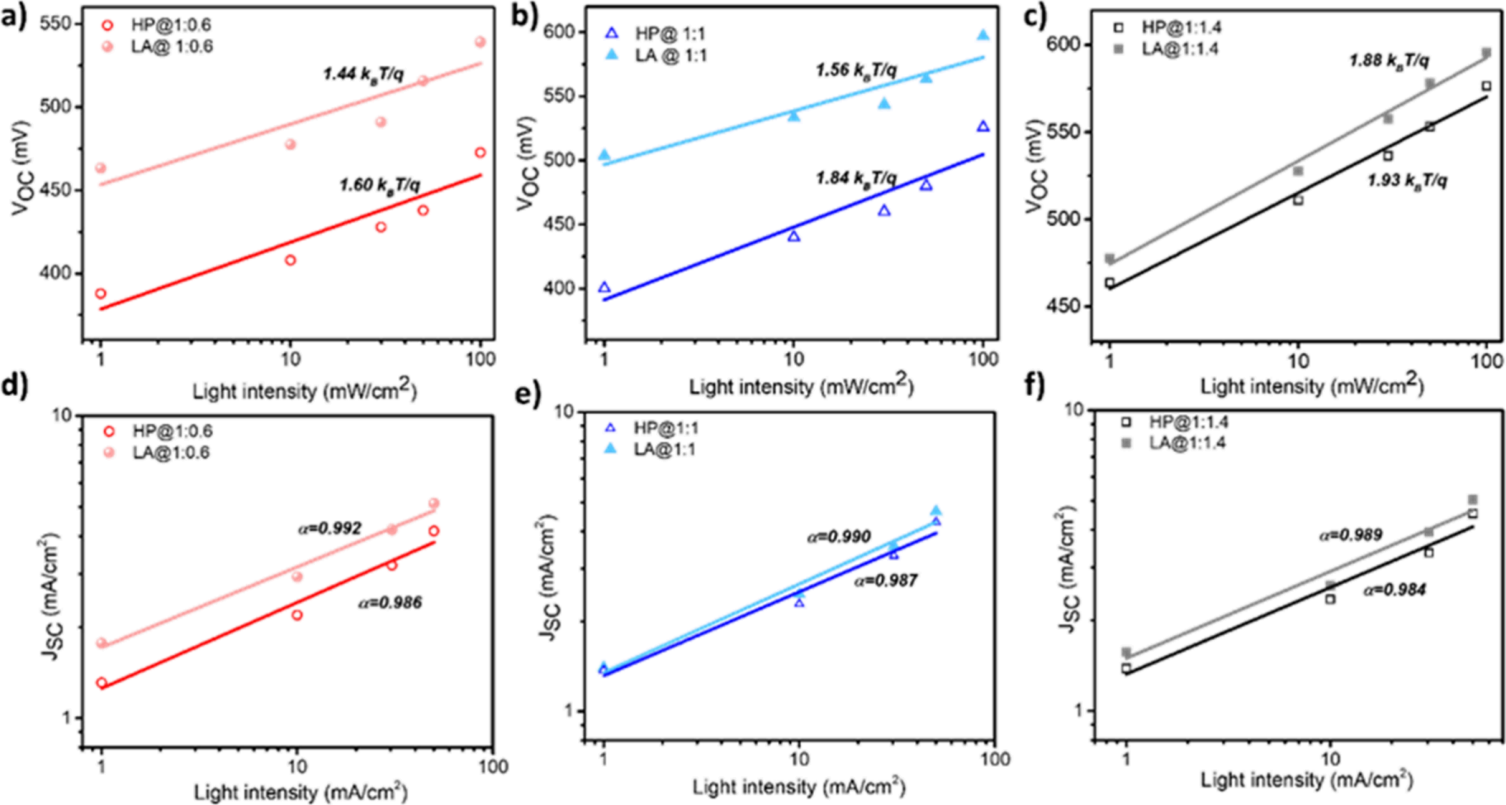
(a–c) Semilogarithmic *V*_OC_–light intensity graphs and (d–f) log–log scale of *J*_SC_–light intensity graphs of P3Ht:PCBM solar cells in different compositions fabricated with HP and LA procedures.

As shown in Figure 5b, *n* values were recorded to be 1.56 and 1.84 *k*_B_ *T*/*q* for LA- and HP-processed P3HT:PCBM (1:1) solar cells, respectively, explaining that trap-assisted recombination is more dominant in these devices fabricated with HP annealing. Figure 5c shows the *n* values of the PCBM-rich blend film created via both annealing processes. 1.88 and 1.93 *k*_B_ *T*/*q* have been calculated for HP and LA-processed solar cells. Both results illustrate that the dominant mechanism is trap-assisted suppressing the efficient charge collection in PCBM-rich devices. Considering these observations, it can be concluded that a reduced PCBM ratio gives rise to reduced trap-assisted recombination in all annealing procedures. Another result is that LA-processed P3HT:PCBM devices in all compositions exhibited improved charge collection. For a deeper analysis of the charge carrier recombination mechanism, we have calculated the α parameter from the slope of the natural logarithm of *J*_SC_ versus the light intensity graph via the power-law dependency (Figure 5d–f). The value of α approaching 1 expresses improved charge extraction and dominant bimolecular recombination.^61^ The obtained values of α were 0.992 and 0.986 for P3HT-rich blend solar cells fabricated with HP and LA procedures, respectively (Figure 5d). Figure 5e represents the calculated α values to be 0.990 and 0.987 for equimolar blend solar cells, and similarly, Figure 5f shows the α results of 0.989 and 0.984 for PCBM-rich blend solar cells via HP and LA processes, respectively.

Both the reduced PCBM ratio in blend P3HT:PCBM solar cells and the annealing type of LA lead to reduced trap density and hence trap-assisted recombination and enhanced device performance. Understanding the electrical conductivity of holes in the P3HT:PCBM blend with different PCBM ratios and annealing procedures, we took advantage of the SCLC method.^62^ It is believed that efficient charge collection is governed by carrier mobilities, which are suppressed by the existence of the traps. Hence, examination of the carrier (electron/hole) mobilities and trap densities of the active layer has a big importance. The hole-only devices with the FTO/PEDOT:PSS/P3HT:PCBM/MoO_3_/Ag configuration have been fabricated. Dark *J*–*V* curves are displayed in Figure 6, and in that graph, three regions need to be characterized. The first region, with the slope value equal to 1 (*m* = 1), is known as the ohmic region. The second region on the graph is the trap-filling limited region; the slope value is bigger than 3 (*m* > 3), introducing a trap-filled area that can be used to calculate the trap density states of devices with the equation *N*_T_ = 2*V*_TFL_εε_0_/*ed* ^2^, where *N*_T_ is the trap density of states, *V*_TFL_ is the trap-filled limit voltage, ε is the dielectric constant of the material, ε_0_ is the dielectric permittivity of vacuum, *e* is the electrical charge, and *d* is the thickness of bulk layer.^63,64^ The third region is called the SCLC region representing a trap-free area, and the slope value is equal to 2 (*m* = 2). In this work, the SCLC mobility was calculated by using Mott–Gurney law (*j* = 9/8εμ*V* ^2^/*d* ^3^, where *J* is the dark current density, ε is the dielectric constant of the material, μ is mobility, *V* is the applied voltage, and *d* is the thickness of P3HT:PCBM layer) with fitting the SCLC region.^65^ The highest hole mobility values of 7.76 × 10^–3^ and 6.61 × 10^–3^ cm^2^/V·s were recorded by the P3HT:PCBM blend with the ratio 1:0.6 annealed via LA and HP procedures, respectively. Afterward, the mobility value of 3.80 × 10^–3^ cm^2^/V·s was obtained by the equimolar blend from the LA-annealed, which is higher than the HP-annealed device mobility of 2.12 × 10^–3^ cm^2^/V·s. The relatively high hole mobility observed in our devices can be attributed to the combined effects of PEDOT:PSS and MoO_3_, which improve hole injection and extraction by enhancing interfacial energetics and reducing transport barriers.^66^ PCBM-rich blend composition exhibited lowered hole mobilities of 2.87 × 10^–3^ and 1.49 × 10^–3^ cm^2^/V·s for both annealing procedures. When we looked at the trap density *N*_T_ of devices, the *N*_T_ of lowest value of 4.63 × 10^15^ cm^–3^ was achieved with LA annealing of P3HT-rich blend (1:0.6). HP-annealed devices with the same blend show a *N*_T_ value of 5.31 × 10^15^ cm^–3^. For the P3HT:PCBM active layer in the equimolar blend, *N*_T_ values were obtained to be 3.60 × 10^15^ and 5.25 × 10^15^ cm^–3^ for HP and LA annealing types, respectively. In the rich active layer (1:1.4) via HP and LA annealing processes, we observed a larger amount of trap densities than the other blend compositions, but the LA procedure still exhibited a smaller *N*_T_ value of 4.93 × 10^15^ cm^–3^ than the value of 9.54 × 10^15^ obtained via the conventional HP annealing procedure. Trap states in P3HT:PCBM blends typically originate from structural defects such as incomplete π–π stacking of P3HT chains, phase separation irregularities, and the presence of chemical impurities or residual solvents. These traps act as recombination centers, limiting the charge carrier lifetime, and our PCE value of 3.10% was obtained, resulting in the overall device performance. In particular, trap density is highly sensitive to the microstructural quality of the BHJ film. LA, applied at a relatively low temperature (100 °C) under illumination, promotes molecular reorganization without causing excessive thermal stress. This process can enhance P3HT crystallinity and improve donor–acceptor phase separation, both of which reduce the density of localized trap states. As observed in our results, LA treatment led to the lowest recorded trap density (4.63 × 10^15^ cm^–3^) for the P3HT-rich (1:0.6) blend, which aligns with the improved carrier mobility and higher device efficiency. This supports the conclusion that LA is an effective and mild strategy for morphological tuning and trap state suppression in organic solar cells. On the other hand, to understand the processes involved in charge transfer within the bulk layer, we investigated the electron mobilities alongside hole mobilities. Electron-only devices were fabricated in the configuration of FTO/TiO_2_/BHJ/Ag, and electron mobilities were obtained using the SCLC method. Afterward, the electron mobilities obtained were compared and analyzed in conjunction with hole mobilities to gain a comprehensive understanding of the mechanism. The results obtained are presented graphically in Figure S7. For the 1:0.6 blend, the calculated electron mobility values of 5.6 × 10^–3^ and 4.87 × 10^–3^ cm^2^/V·s were obtained via annealing with LA and HP procedures, respectively. The electron mobility values of 3.24 × 10^–3^ and 1.77 × 10^–3^ cm^2^/V·s were recorded for the 1:1.4 blend ratio with LA- and HP-annealed devices. For the equimolar ratio blend with LA and HP annealing, the electron mobility values were calculated to be 4.47 × 10^–3^ and 2.63 × 10^–3^ cm^2^/V·s, respectively. Similar to hole mobilities, PCBM-rich devices illustrated lowered electron mobilities for both annealing types. According to these results, we have two findings: (1) Increased P3HT ratio in the blend gives rise to higher hole/electron mobility representing favorable charge collection along the vertical direction of an active layer via donor and acceptor pathways in the device. (2) The LA-annealing procedure improves the hole/electron mobility in all P3HT:PCBM blends in different ratios because of decreased defect density and charge carrier recombination, which confirms the output solar cell performance. The comparative analysis of hole and electron mobilities clearly indicates that the charge-transport balance strongly correlates with the device performance. Among all studied compositions, the P3HT-rich blend (1:0.6) annealed via LA exhibited the most balanced and highest hole mobility (7.76 × 10^–3^ cm^2^/V·s) along with a relatively low trap density (4.63 × 10^15^ cm^–3^), which collectively contributed to the highest device efficiency (PCE = 3.10%). Conversely, the PCBM-rich blend (1:1.4), especially under HP annealing, showed a significant imbalance due to drastically reduced hole mobility (1.49 × 10^–3^ cm^2^/V·s), higher trap density (9.54 × 10^15^ cm^–3^), and ultimately lower device performance. The comparison between electron and hole mobilities obtained from SCLC measurements suggests that devices with better-matched mobilities exhibit enhanced charge extraction and reduced space charge accumulation, leading to higher FF and *J*_SC_. In contrast, the mobility imbalance increases carrier recombination, limits charge collection, and results in performance losses. These findings highlight the importance of optimizing not only the absolute carrier mobilities but also their ratio through careful control of blend composition and annealing treatment.^3^

**Figure 6:**
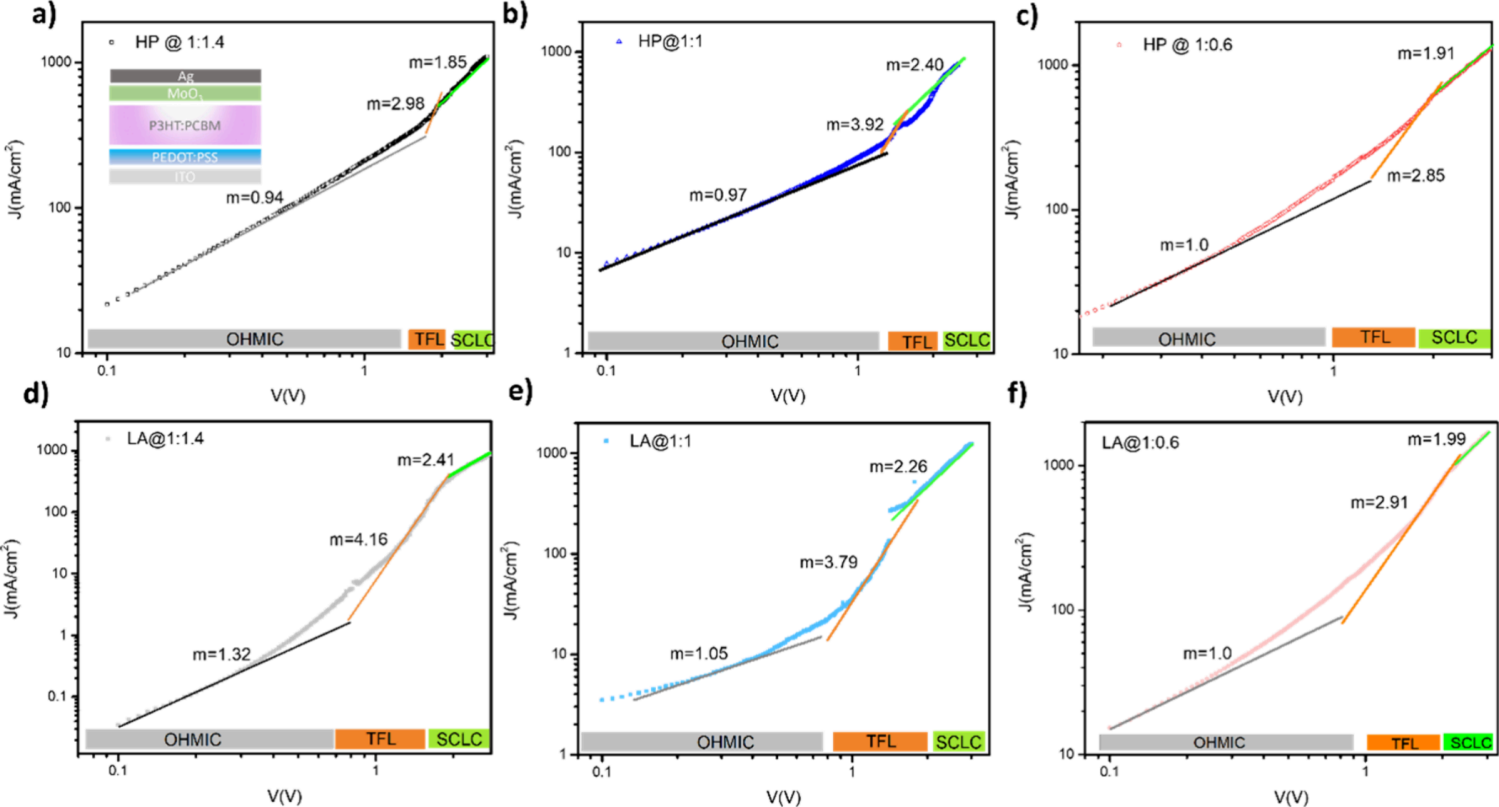
SCLC results of hole-only devices with the configuration of ITO/PEDOT:PSS/P3HT:PCBM/MoO_3_/Ag. log–log dark *J*–*V* graphs designated with OHMIC, TFL, and SCLC regions of P3HT:PCBM blend in (a, d) 1:1.4, (b, e) 1:1, and (c, f) 1:0.6 ratios annealed via HP and LA process, respectively.

## Conclusions

In conclusion, LA annealing has been applied to P3HT and P3HT:PCBM blend films to investigate the effect on film morphology and formation for different annealing temperatures (from 100 to 120 °C) and annealing times (5, 10, and 15 min). The LA-annealed P3HT films illustrated better crystallization of P3HT films even in short durations, and it was even found to yield superior results, enabling better crystallization and resulting in reduced trap sites when compared to the HP method. Then, the impact of annealing as a treatment process was examined on the optical, morphological, and electrical properties of the P3HT:PCBM films used as the active layer in organic solar cells. According to detailed results, the optimum annealing procedure is determined as 100 °C for 15 min. The best performance has been observed by the LA-annealed P3HT:PCBM (1:1) device with a PCE of 3.10%, *J*_SC_ of 9.11 mA/cm^2^, *V*_OC_ of 610 mV, and FF of 0.55. To further clarify that the positive outcomes of the LA-annealing process could be attributed to P3HT, a study of the varied P3HT:PCBM blend ratios (1:0.6, 1:1, and 1:1.4) was conducted. It was observed that both the traditional HP method and the LA method yielded better results in mixtures in which the P3HT domain was rich. However, in mixtures in which the PCBM domain was rich, there was a relatively smaller performance difference between films obtained through the HP and LA methods, whereas the LA procedure outperformed in mixtures in which P3HT was in excess. These findings underscore two significant conclusions: first, an increased P3HT ratio in the blend contributes to higher hole mobility, facilitating favorable charge collection along the vertical direction of the active layer through donor and acceptor pathways in the device. Second, the LA-annealing procedure plays a crucial role in improving hole mobility across all P3HT:PCBM blends with different compositions, attributed to the reduction in the defect density and charge carrier recombination, thereby affirming enhanced solar cell performance. Considering this information, it can be said that the LA method has the potential to enhance the performance of P3HT:PCBM films used as the active layers in organic solar cells. Our findings establish LA annealing as a scalable, low-cost, and efficient post-treatment method for optimizing active layer morphology and boosting performance in polymer-based solar cells. This work paves the way for the broader adoption of light-assisted annealing techniques in solution-processed optoelectronic device manufacturing.

## Experimental Section

### Materials

Fluorine-doped tin oxide (FTO) substrates (2.5 × 2.5 cm^2^, 14 Ω cm^–1^) were purchased from OPV Tech. 2-Propanol (for HPLC grade-99.9%), hydrochloric acid (HCL), titanium isopropoxide (TiOPr_4_), anhydrous chlorobenzene (CB), P3HT, and molybdenum­(VI) oxide (MoO_3_) (99.97%-trace metal basis) were purchased from Sigma-Aldrich. PCBM was bought from Solenne. Poly­(3,4-ethylenedioxythiophene) polystyrenesulfonate (PEDOT:PSS) was obtained from Heraeus Clevios P VP Al4083. All of the purchased materials were used without any further purification.

### Device Fabrication

First, FTO substrates were cleaned with an ultrasonic cleaning bath for 15 min with a detergent, deionized water, acetone, and isopropanol, sequentially. The cleaned substrates were submitted to oxygen plasma treatment for 7 min to remove any remaining impurities. Titanium dioxide precursor solution (5.6 mL of isopropyl alcohol, 35 μL of hydrochloric acid, and 369 μL of titanium­(IV) isopropoxide) was prepared and coated by the spin coating method to form c-TiO2. The compact layer TiO_2_ was formed by annealing at 460 °C for 1 h. After being cooled down to 100 °C, the films were immediately taken to the N_2_-filled glovebox to deposit the P3HT:PCBM BHJ layer. The P3HT:PCBM BHJ solution was prepared by dissolving 10 mg of P3HT in 1 mL of CB (%1) and 10 mg of PCBM in 1 mL of CB(%1), separately. Different ratios of P3HT:PCBM blends were prepared (1:0.6, 1:1, and 1:1.4). The BHJ layer was coated by a one-step spin coating at 2000 rpm for 45 s. P3HT, PCBM, and P3HT:PCBM thin films were coated with a spin-coater in the same way. After that, thin films are annealed via (HP) and (LA) methods. HP annealing was done by a conventional HP in a N_2_-filled glovebox at different temperatures and durations. LA annealing was done in a box equipped with an OSRAM 64,652 HLX-Halogen lamp (Max Power of 250 mW/cm^2^), and temperature was controlled with an electronic card and monitored via a PT100 thermocouple. The thermocouple, which is integrated within the device, was calibrated by the manufacturer to accurately reflect the surface temperature. The system’s temperature can be adjusted via the control unit, and it is displayed in Celsius. The LA and HP systems are illustrated inSupporting Information­( Scheme S1). Finally, 8 nm MoO_3_ and 100 nm Ag contacts were deposited under vacuum of 2 × 10^–7^ Torr by a glovebox-integrated thermal evaporator.

### Device Characterization

A Keithley-2400 source meter was used to record the current density–voltage (*J*–*V*) curves of the BHJ solar cells under a simulated glovebox (AM1.5G, 100 mW/cm^2^). Before each test, the light intensity was calibrated using a verified Si reference solar cell. An Enlitech QE-R measuring equipment was used to calculate the IPCE. An Edinburgh Instruments 655 nm laser was used to record the steady-state PL and TRPL spectra of the BHJ films placed on top of the glass substrate. The PL measurements were carried out utilizing a 625 OR 450-W Xe arc lamp as an optical excitation source and monochromatic light as an excitation source. The surface morphology of the thin films was obtained using AFM (Ambios model). A Rigaku Ultima IV diffractometer was used to measure the crystallinity and purity of the annealed organic thin films. An UV–vis absorption spectrophotometer (PerkinElmer Lambda 950) was used to measure the absorption spectra.

## Supplementary Material



## Notes

Experimental setup of HP and LA annealing systems; AFM images of the P3HT thin film; P3HT:PCBM blend films in different compositions; XRD results of P3HT:PCBM blends; statistical chart graphs of *V*_oc_ values for P3HT:PCBM HP- and LA-annealed devices; and calculated trap density and mobility results for P3HT:PCBM devices (PDF)

## ‡

Helmholtz-Zentrum Berlin, Department of Active Materials and Interfaces for Perovskite Solar Cells (SE-AMIP), Albert-Einstein-Straße 12, 12489 Berlin, Germany
