# Supplementary material for: Boosting Organic Solar Cell Performance via Light-Assisted Crystallization of P3HT:PCBM Blend
Source: ACS Omega. 2025 Jul 23;10(30):33341–52. doi: 10.1021/acsomega.5c03436 (PMC12332790; doi:10.1021/acsomega.5c03436)
Supplement: Supplementary file 1 [file ao5c03436_si_001.pdf]

Supporting Information

Boosting Organic Solar Cell Performance via Light-Assisted Crystallization of P3HT:PCBM Blend

Duygu Akın Kara[*a†], *Sevdiye Basak Turgut* [a†], *Burak Gultekin*[*a].

[a]Ege University, Solar Energy Institute, 35000, Izmir, Turkey.

KEYWORDS P3HT:PCBM, bulk heterojunction, solar cell, film crystallization, recombination

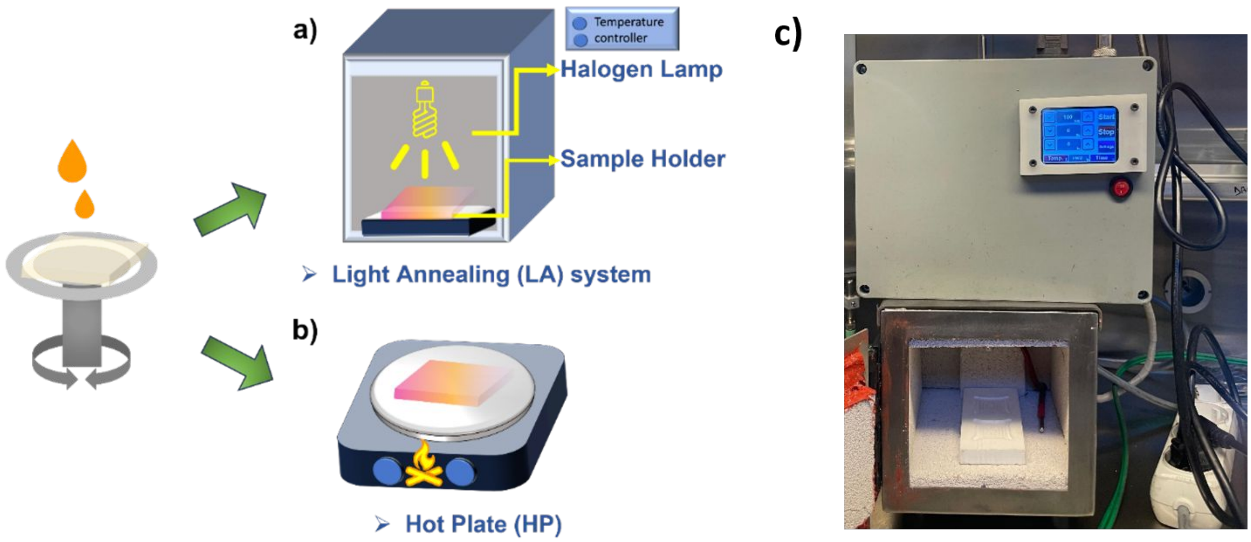

Scheme S1: Schematic representation of a) LA system, b) HP system, and c) the photo of glove-box integrated LA system.

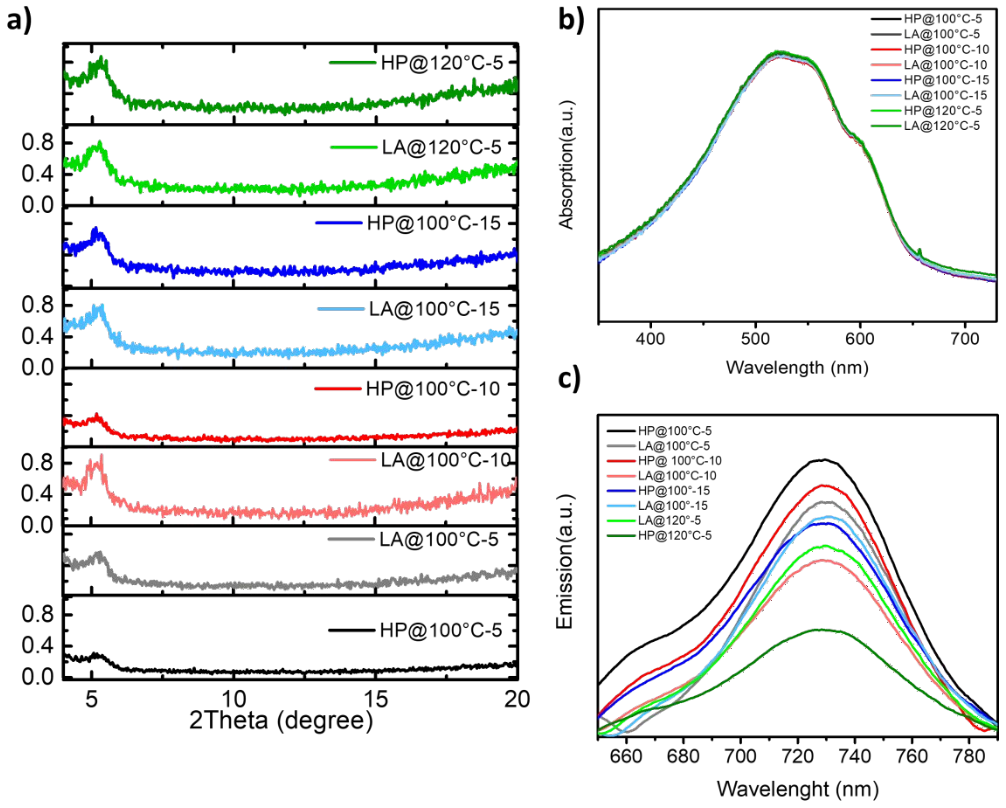

Figure S1. a) X-ray diffraction, b) UV-Vis absorption and c) Photoluminescence (PL) spectrum of P3HT thin films annealed at various temperature and time with different annealing procedure (HP: Hot-plate, LA: Light-Assisted).

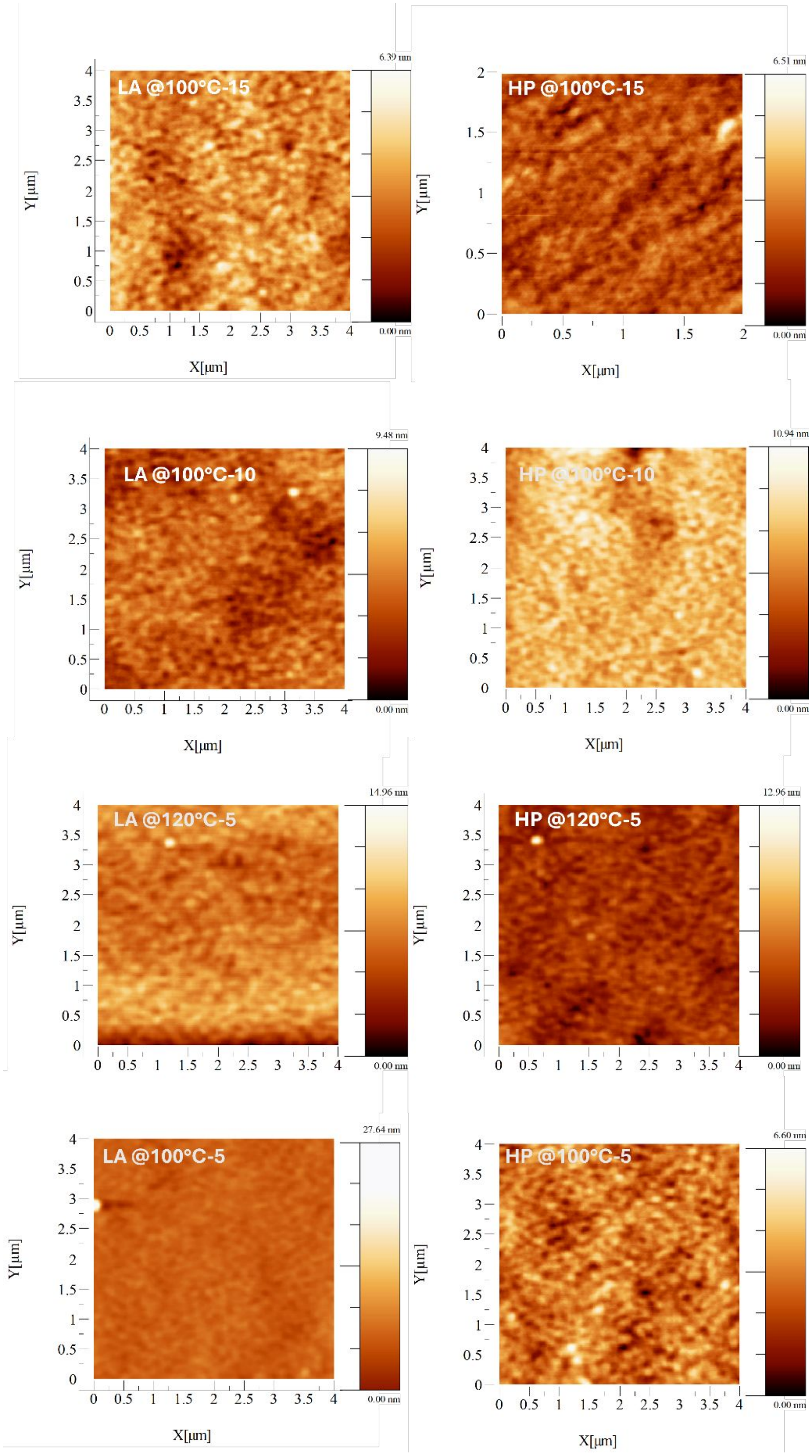

Figure S2. AFM images of P3HT (%1 wt.) films on glass, annealed via HP and LA annealing procedure including different temperature and duration.

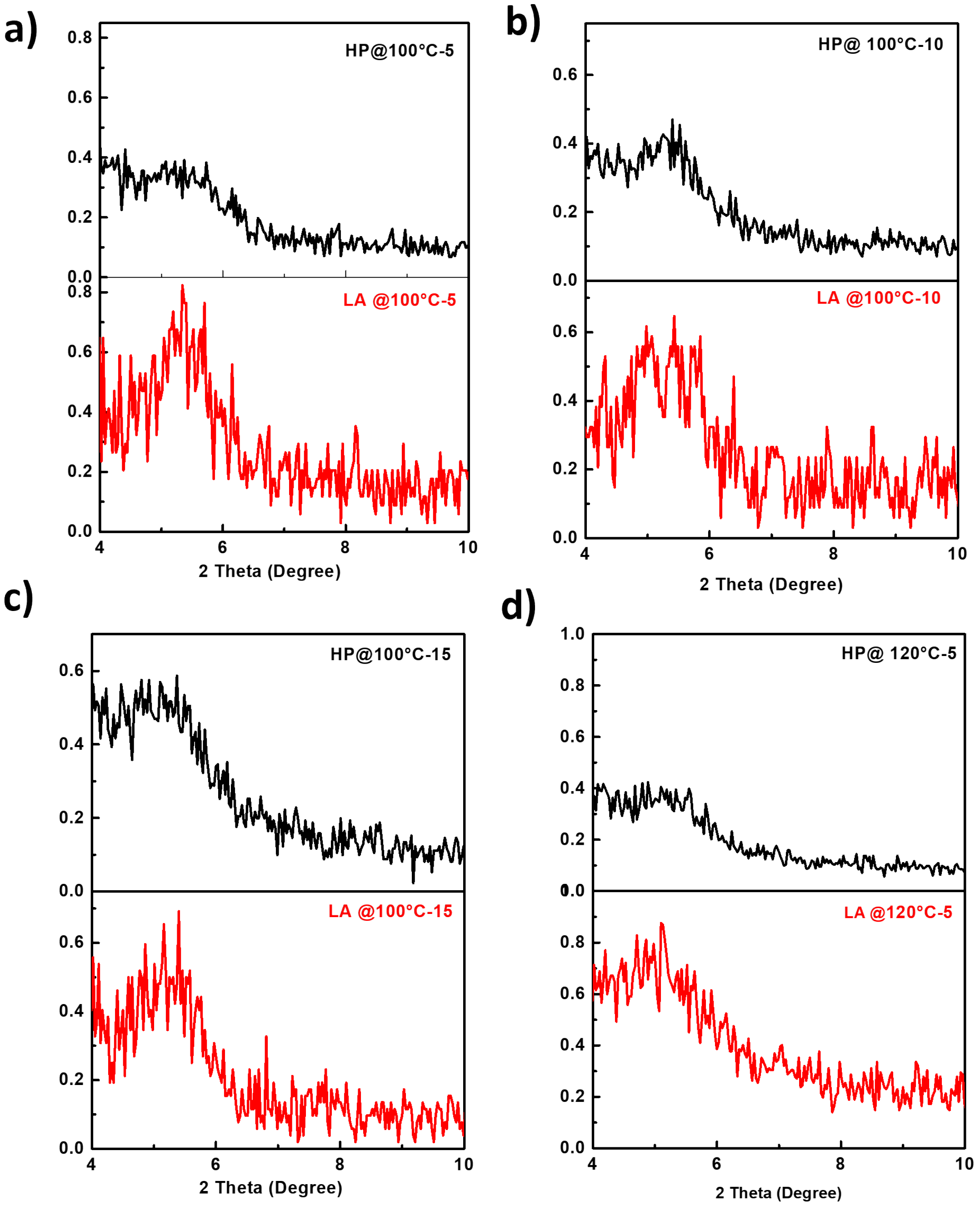

Figure S3. X-ray diffraction spectrum of P3HT:PCBM thin films annealed at various temperature and time with different annealing procedure (HP: Hot-plate, LA: Light-Assisted). a) 100˚ C for 5 min, b) 100˚ C for 10 min, c) 100˚ C for 15 min and d) 120˚ C for 5 min.

Table S1. Summary of the effect of various annealing methods and conditions on the grain size.

Sample	Annealing Condition	Crystallite Size (nm)
LA@120°C–5	Light annealing, 120 °C, 5 min	5.39
HP@120°C–5	Hot plate annealing, 120 °C, 5 min	6.45
HP@100°C–15	Hot plate annealing, 100 °C, 15 min	7.93
LA@100°C–15	Light annealing, 100 °C, 15 min	10.87
HP@100°C–10	Hot plate annealing, 100 °C, 10 min	9.12
LA@100°C–10	Light annealing, 100 °C, 10 min	6.72
HP@100°C–5	Hot plate annealing, 100 °C, 5 min	6.66
LA@100°C–5	Light annealing, 100 °C, 5 min	8.18

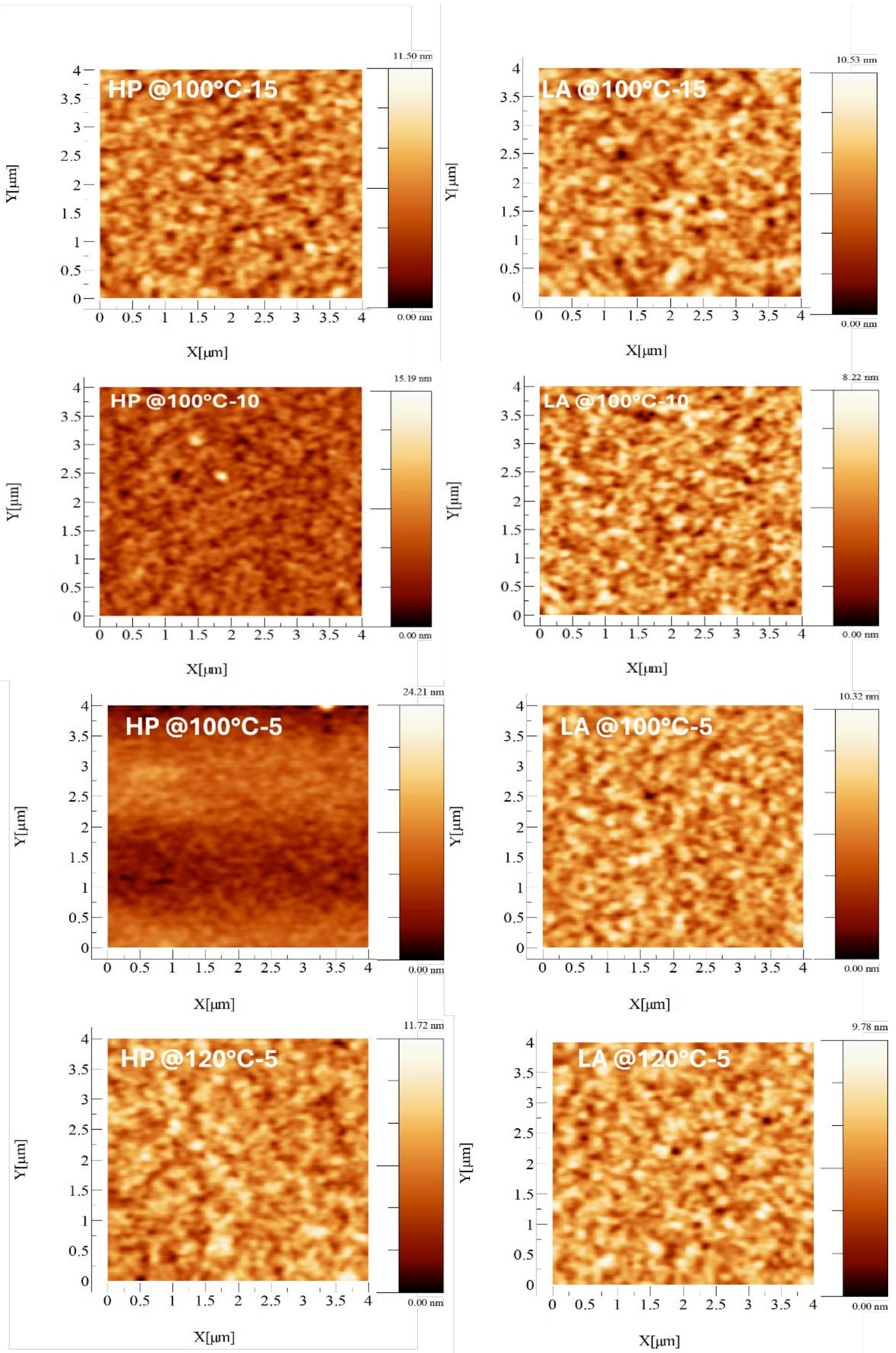

Figure S4. AFM images of P3HT:PCBM (1:1) films on glass, annealed via HP and LA procedure including different temperature and duration

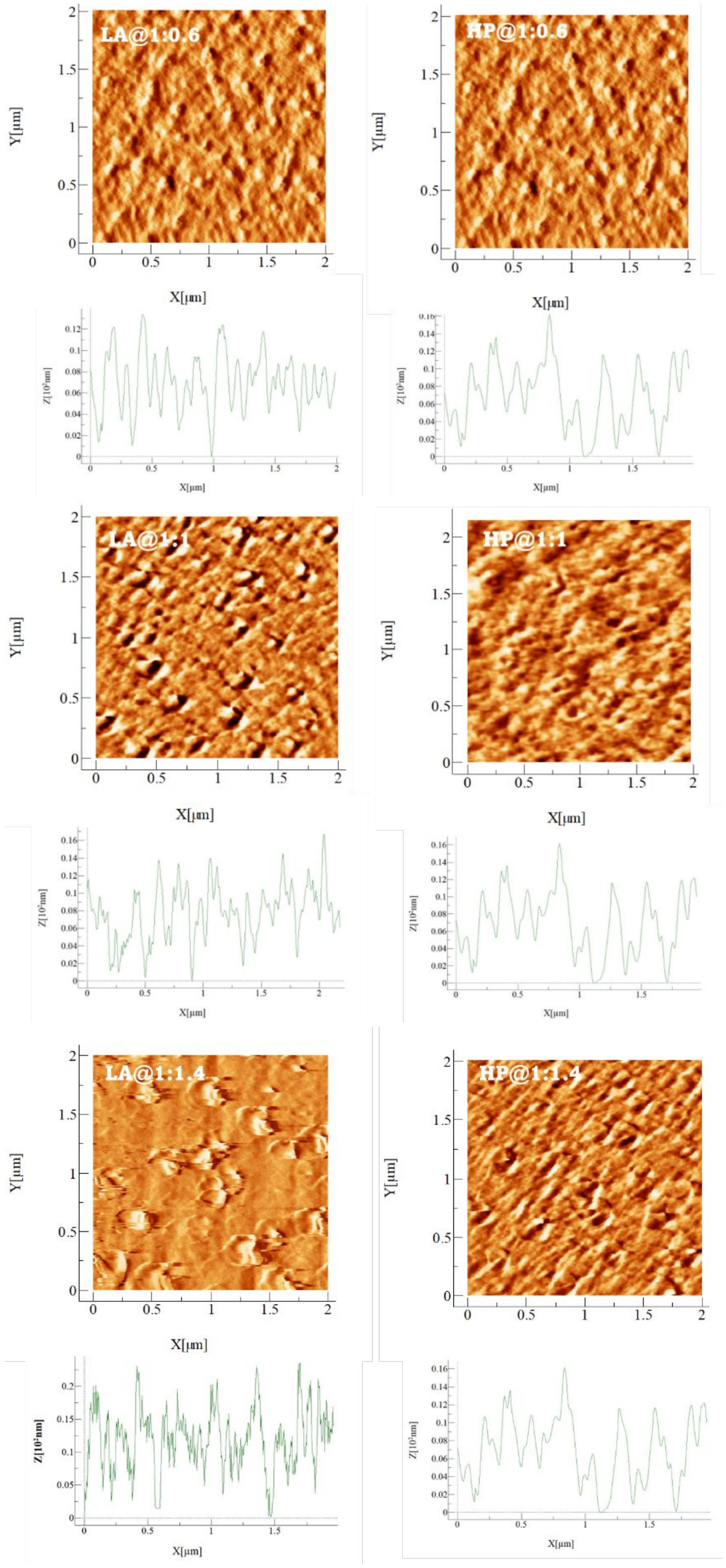

Figure S5. AFM images of P3HT:PCBM films with varied ratio on glass, annealed via HP and LA procedure

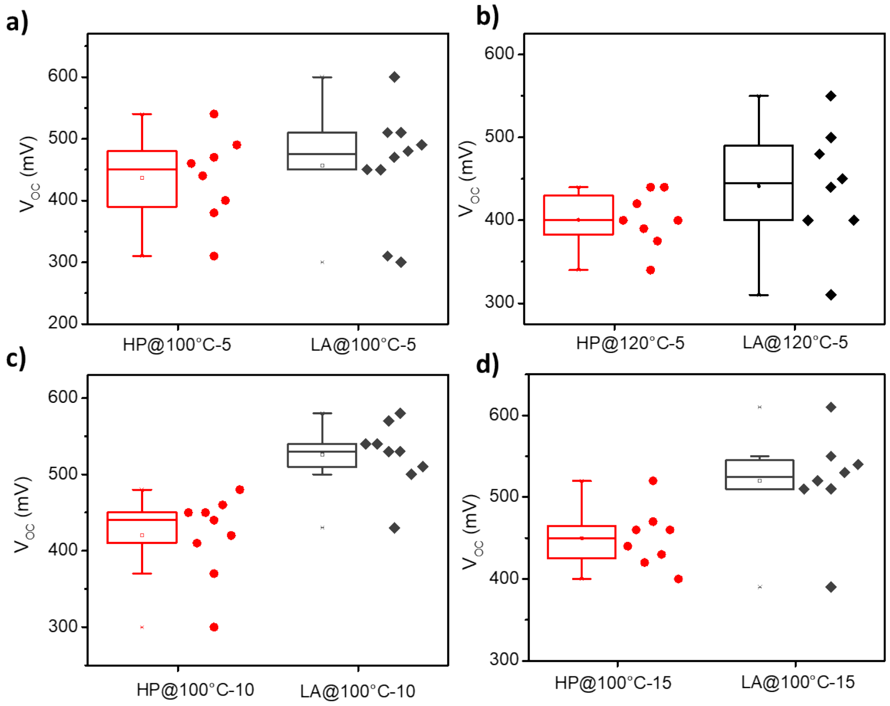

Figure S6. a) 100˚ C for 5 min, b) 120˚ C for 5 min, c) 100˚ C for 10 min and d) 100˚ C for 15 min of statistical chart graphs of V_{OC} device parameters for P3HT:PCBM devices annelaed HP and LA procedures.

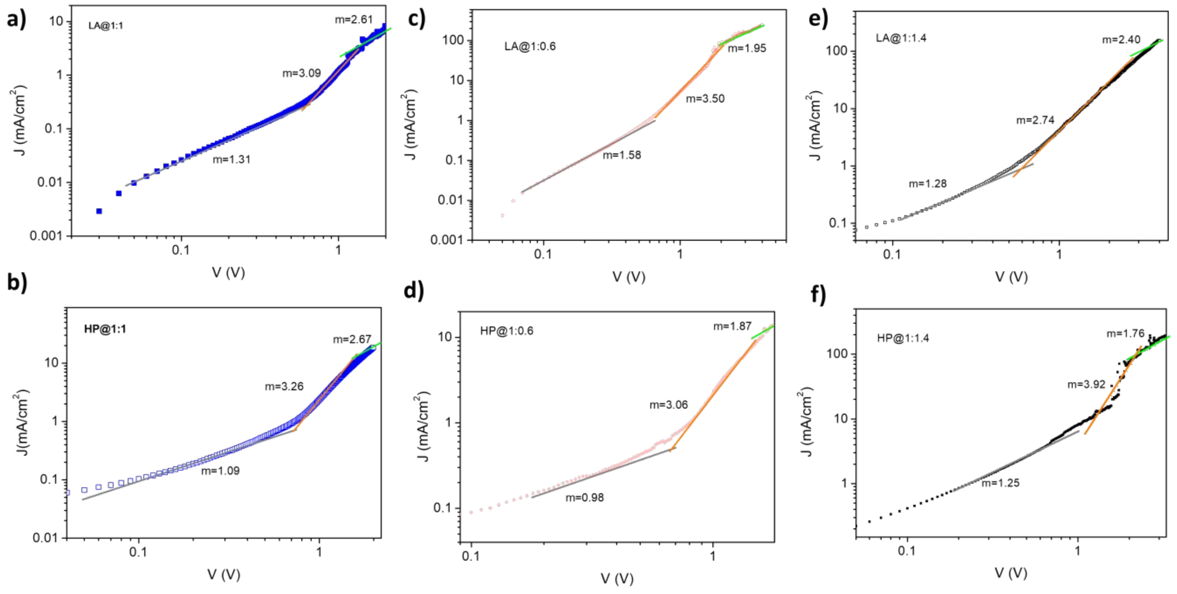

Figure S7. Space charge Limited Current (SCLC) results of electron only devices with the configuration of ITO/TiO2/P3HT:PCBM/Ag. log-log dark J-V graphs designated with OHMIC, TFL and SCLC regions of P3HT:PCBM blend in (a-b) 1:1 ratio, (c-d) 1:0.6 ratio, (e-f) 1:1.4 ratio annealed via LA and HP process, respectively.
